# Claudin-3 Loss of Expression Is a Prognostic Marker in Castration-Resistant Prostate Cancer

**DOI:** 10.3390/ijms24010803

**Published:** 2023-01-02

**Authors:** María J. Orea, Javier C. Angulo, Ana González-Corpas, David Echegaray, Marcos Marvá, María V. T. Lobo, Begoña Colás, Santiago Ropero

**Affiliations:** 1 Departamento de Biología de Sistemas, Unidad Docente de Bioquímica y Biología Molecular, Universidad de Alcalá, 28805 Alcalá de Henares, Spain; 2 Departamento Clínico, Facultad de Ciencias Médicas, Universidad Europea, 28005 Madrid, Spain; 3 Servicio de Urología, Hospital Universitario de Getafe, 28907 Madrid, Spain; 4 Departamento de Física y Matemáticas, Unidad Docente de Matemáticas, Universidad de Alcalá, 28805 Alcalá de Henares, Spain; 5 Departamento de Ciencias Biomédicas I, Biología Celular y Genética, Universidad de Alcalá, 28805 Alcalá de Henares, Spain; 6 Instituto Ramón y Cajal de Investigaciones Sanitarias (IRYCIS), 28034 Madrid, Spain

**Keywords:** prostate, castration-resistant prostate cancer, DNA methylation, CLDN3, prognosis

## Abstract

Castration-resistant prostate cancer (CRPC) development is the foremost concern after treatment of patients with high risk with locally advanced or metastatic prostate cancer. Androgen receptor (AR) is the main driver of CRPC development, through its interaction with epigenetic modifier genes, placing epigenetics modifications in the forefront of CRPC development. Comparing the DNA methylation and expression profile of androgen-sensitive and -refractory prostate cancer cells, we describe the epigenetic silencing of claudin-3 (CLDN3) in AR positive cells resistant to androgen deprivation (LNCaP-abl). CLDN3 silencing was associated with DNA methylation, loss of histone acetylation and H3K27 methylation, and was re-expressed by the combined treatment with the epigenetic modulators Aza and SAHA. From a functional point of view, CLDN3 loss was associated with increased cellular invasion. Immunohistochemical analysis showed decreased CLDN3 expression in samples from CRPC patients. Interestingly, CLDN3 expression was significantly decreased in samples from patients with high total Gleason score (≥8) and locally advanced tumors. Finally, CLDN3 loss of expression was associated with worse disease-free survival and time to clinical progression. In conclusion, our findings strongly indicate that epigenetic silencing of CLDN3 is a common event in CRPC that could be useful as a molecular marker for the prognosis of prostate cancer patients and to discriminate aggressive from indolent prostate tumors.

## 1. Introduction

Prostate cancer is the most frequent cancer in males and the second-leading cause of male cancer-death [1]. Prostate cancer cell growth and survival is dependent on androgens and androgen receptor, allowing their use as a therapeutic target for advanced, metastatic, or recurrent prostate cancer [2]. However, disease progression, despite antiandrogen therapy, is the rule, with a transition from androgen-dependence to castrate-resistance, in which cancer cells become driven by alternative growth signaling pathways [3]. This makes mandatory the investigation of new forms of therapy, including theranostic agents targeting neuroendocrine prostate cancer [4,5,6,7], the aggressive histologic subtype of prostate cancer that most commonly arises in later stages of prostate cancer as a mechanism of treatment resistance [3,8].

Despite the depletion of circulating androgens after androgen-deprivation therapy (ADT), sustained AR signaling remains the primary molecular mechanism driving CRPC [9,10]. Several mechanisms have been proposed for the persistent AR activity observed in CRPC, including AR amplification and overexpression [11,12], AR mutations [13], intratumoral androgen synthesis [14], overexpression of AR coactivators [15], aberrant kinase pathway activation [16], and the constitutive expression of AR splice variants [17]. These molecular findings allowed the development of new therapies to block AR activity and androgen synthesis. Treatment options for CRPC patients include abiraterone, which inhibits a pivotal enzyme in androgen synthesis, or enzalutamide, which is an antagonist of AR [18,19]. However, resistance to the second-generation hormonal treatments has emerged in the last years. The only treatment option for these patients is traditional chemotherapeutic agents, such as docetaxel or cabazitaxel, but unfortunately these treatments only increase patient survival a few months [20]. Thus, the study of the molecular mechanism underlying resistance to ADT is a priority health issue, which could allow the development of personalized therapies.

AR regulates gene expression through its interaction with co-activators and co-repressors at the regulatory sequences. These coregulators include epigenetic modifiers that regulate epigenetic modifications and then chromatin structure around the androgen responsive elements (AREs) affecting AR transcriptional activity. The best-known epigenetic modifications so far are DNA methylation and posttranslational histone modifications. A growing number of epigenetic modifying enzymes have been found as co-regulators of AR function that have contrary effects on gene expression. AR interacts with the histone acetyltransferases (HATs) p300/CBP and p160/SRC, causing an increase in gene expression, but it also binds to corepressor complexes containing histone deacetylases (HDACs) [21,22], which determines the deacetylation of histones and a more condensed state of the chromatin that impedes the access of the transcriptional machinery. In addition, AR also modifies histone methylation at regulatory sequences by recruiting the histone methyltransferases (HMTs) and demethylases (HDMs).

It has been suggested that AR regulates a different gene expression profile in hormone-sensitive prostate cancer and CRPC [23]. These results could be explained, at least in part, by changes in the epigenetic modifications, DNA methylation, and histone modifications at the gene regulatory regions induced by changes in AR-coregulator interaction pattern in androgen-dependent prostate cancer and CRPC.

In previous studies, we have identified groups of hypermethylated genes to be used as molecular markers for prostate cancer diagnosis and for the prognosis of advanced prostate cancer [24,25]. In the present study, by combining genome-wide DNA methylation and expression analysis we have identified a set of epigenetically deregulated genes with prominent functions in CRPC development. Among these, we have selected CLDN3 that belong to a family of integral membrane proteins that are part of tight junctions. These intercellular junctions act as permeability barriers conferring polarity to epithelial cells and playing a crucial role in maintaining the cell–cell integrity. Unraveling the molecular mechanism that controls its expression is crucial, since claudins deregulation has been correlated with cancer progression and metastasis [26].

## 2. Results

### 2.1. DNA Methylation Profile of Androgen-Independent Prostate Cancer Cells

The aim of this study was to unmask epigenetically deregulated genes in CRPC as potentially useful as molecular markers. To mimic the properties of the androgen-sensitive forms of the disease and CRPC, we utilized LNCaP, LNCaP abl (abl), PC3, and DU145 cell lines. LNCaP is an androgen-sensitive cell line that represents the androgen-sensitive prostate cancer, and abl is its androgen-refractory counterpart, which are AR-positive and represent most of the CRPC. Abl cells were generated after long-term androgen deprivation of the LNCaP cell line [27]. Consistent with its CRPC phenotype, abl cells grow faster than the parental cell line LNCaP cells in hormone-depleted medium [28,29], and did not respond to enzalutamide treatment (Figure S1). PC3 and DU145 represent a small proportion (20%) of CRPC that do not express AR.

**Figure 1:**
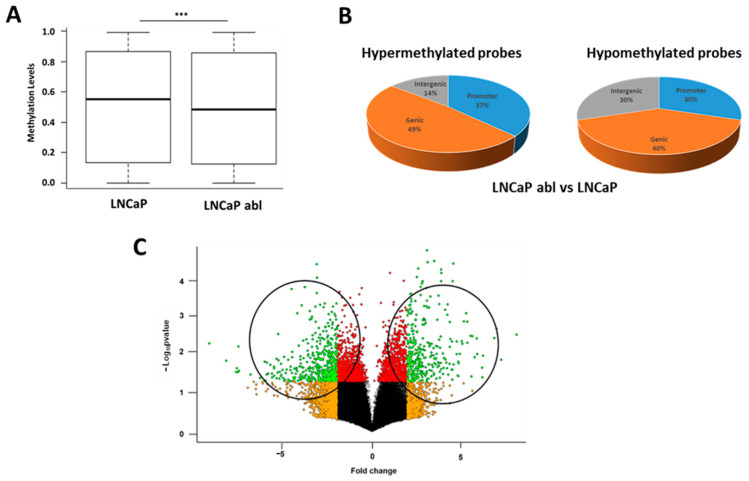
DNA methylation and expression profile of LNCaP and LNCaP abl cell lines. (**A**) Comparison of global DNA methylation levels of LNCaP and LNCaP abl cell lines. (**B**) Venn diagram showing genomic location of hypermethylated and hypomethylated probes in LNCaP abl cells compared to LNCaP cell line. (**C**) Volcano plot comparing gene expression profile between LNCaP and LNCaP abl cell lines. Each data-point represents a gene, with green dots representing downregulated (fold change < −2 and *p* value < 0.05) and upregulated (fold change > 2 and *p* value < 0.05). *** *p* < 0.001.

To identify deregulated genes that might account for progression of CRPC maintaining AR function, we performed a genome-wide measurement of DNA methylation status of ~480.000 CpGs using the Illumina Infinium Human Methylation 450K with the cell lines described previously. Following normalization and batch correction of array data, first we performed differential methylation analysis between abl and LNCaP cells revealing a significant decrease in DNA methylation in abl cells (Figure 1A), as was expected, since global DNA hypomethylation has been extensively associated with prostate cancer progression. To identify differential methylated probes, we selected those with a ∆β > 0.5 between LNCaP and abl cells. With this criterion, we found 1116 probes hypermethylated and 2594 hypomethylated in abl respect to LNCaP cells. Among these, 37% of the hypermethylated probes were located in the promoter region of 264 genes, while 30% of the hypomethylated were found in the promoter region of 477 genes. By contrast, the number of hypomethylated probes located in intergenic regions was greater than the hypermethylated (Figure 1B).

Since the main objective of this study was to find deregulated genes by DNA methylation changes in CRPC maintaining AR expression and function, we next compared the methylation profile of AR negative (PC3 and DU145) and LNCaP cells, and lists of hypo- and hypermethylated genes were selected following similar criteria described for abl cells. The comparison of the differentially methylated genes in abl and AR-negative cells will allow us to discriminate those changes that occur because of the loss of AR function from those that occur in tumors maintaining AR function.

This comparison revealed genes hypermethylated and hypomethylated, respectively, in both abl and AR negative cells, indicating that those could be associated with CRPC progression independently of the AR function. However, more interesting are the specific differentially methylated genes in abl because this cell line recapitulates the CRPC expressing AR that represent the majority of the CRPC.

To further establish a correlation between DNA methylation and gene expression of the differentially methylated genes only in abl, we re-analyzed gene expression profiles of LNCaP and abl cells already published [17] (GSE11428) (Figure 1C) and compared them to the specific DNA methylation profile of abl cells. We found 22 genes that were hypermethylated and downregulated and 22 genes that were hypomethylated and upregulated in abl compared with LNCaP cells (Table S1). Among these, we selected one hypermethylated (*CLDN3*) and two hypomethylated (*EGF* and *ELF5*) genes for further validation.

**Figure 2:**
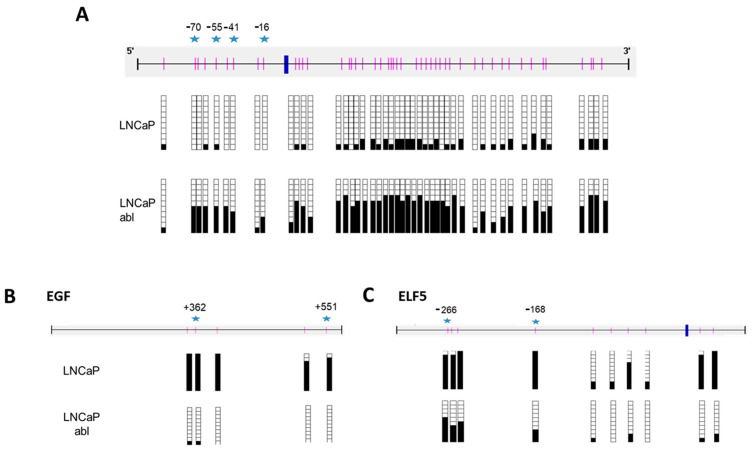
Analysis of CLDN3 (**A**), EGF (**B**), and ELF (**C**) methylation status in LNCaP and LNCaP abl cell lines. Schematic depiction of the CLDN3 (**A**), EGF (**B**), and ELF (**C**) promoter region around the corresponding transcription start site (thick blue vertical line). CpG dinucleotides are represented as short vertical lines. Results of bisulfite genomic sequencing of 10 individual colonies are shown. Presence of a methylated or unmethylated cytosine is indicated by a black or white square, respectively. Blue stars represent the CpGs analyzed in the array.

Bisulfite sequencing confirmed the data from the methylation array for *CLDN3*, *EGF*, and *ELF5* (Figure 2). In particular, we observed a high correlation between the data from the array and bisulfite sequencing, as shown in the β-values for some of the CpGs analyzed in the array (Table S2) and the percentage of methylated clones for the same CpG.

**Figure 3:**
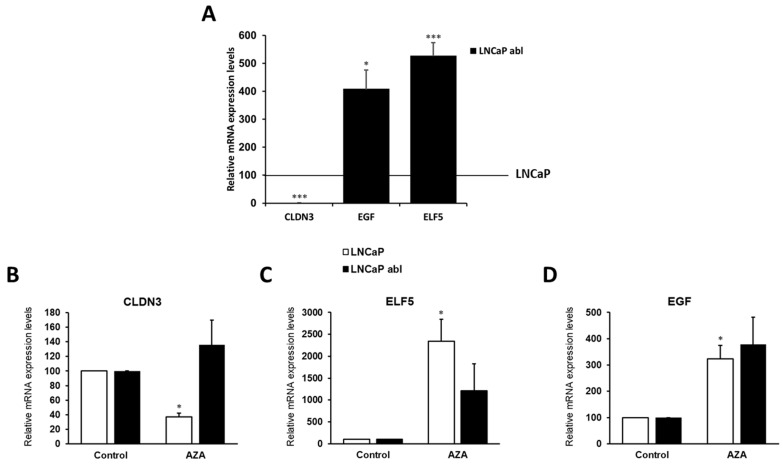
QRT-PCR showing the mRNA expression levels of CLDN3, EGF, and ELF5 in prostate cancer cell lines (**A**). The treatment with 2 μΜ (AZA) of the demethylating agent 5-aza-2-deoxycytidine reactivates expression in methylated cell lines (**B**–**D**). Data shown represent the mean  ±  s.e.m. of three independent experiments completed in triplicate. * *p* < 0.05; *** *p* < 0.001.

The correlation between DNA methylation and expression of the selected genes was confirmed by qRT-PCR. CLDN3 mRNA were almost absent in abl cells (Figure 3A), and the treatment with the demethylating agent 5-aza-2-deoxycytidine (aza) increased its levels, indicating that the observed decrease in its expression are due, at least in part, to DNA promoter methylation (Figure 3B). However, aza treatment decreased CLDN3 mRNA in LNCaP cells, where unmethylated, that could be due to changes in the expression of transcription factors regulating CLDN3 expression. The levels of EGF and ELF5 mRNA were higher in abl cells correlating with promoter hypomethylation (Figure 3A), but the treatment with aza increased their expression (Figure 3C,D). This effect could be due to changes in the expression of transcription factors regulating their expression or reactivation of additional transcription start sites present in the body of the genes. As was expected, EGF and ELF5 levels were increased in the cell methylated cell line (LNCaP) after aza treatment (Figure 3C,D).

### 2.2. Loss of CLDN3 Increases Cell Invasiveness

From the selected genes, we focused on *CLDN3*. This gene codifies for a protein that belongs to a family of claudins, which are important components of the tight junctions (TJs) and are crucial structures for the maintenance of cellular polarity in epithelial and endothelial cells [30]. The expression profile of CLDN3 can be tissue-specific and vary among cell types within a given tissue, suggesting a specific role of CLDN3 in different tissue or different environment. We observed that CLDN3 was silenced in the cell line recapitulating a more aggressive and therapy-resistant stage of prostate tumors (abl), but it was expressed in androgen sensitive prostate cancer cells (LNCaP), representing initial stages of prostate cancer development.

**Figure 4:**
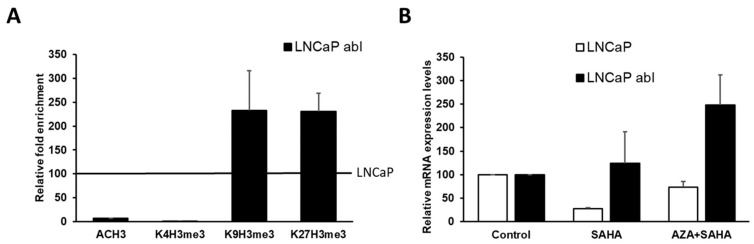
(**A**) Chromatin immunoprecipitation analyses for histone H3 acetylation (AcH3) and the methylation of lys4 (K4H3me3), lys9 (K9H3me3), and lys27 (K27H3me3) of histone H3 of CLDN3 in prostate cancer cell line. The graph shows the enrichment of CLDN3 promoter DNA measured by real-time PCR. Data shown represent the mean  ±  s.e.m. of two independent experiments. (**B**) QRT-PCR showing mRNA expression levels of CLDN3 in prostate cancer cell lines treated with 5 μM SAHA or 2 μΜ AZA plus 5 μM SAHA. Data shown represent the mean  ±  s.e.m. of three independent experiments completed in triplicate.

The data showed on Figure 2 and Figure 3 established a link between *CLDN3* promoter hypermethylation and gene silencing. However, CLDN3 re-expression observed after demethylating treatment was smaller than could be expected. This lack of effect could be due to the presence of additional epigenetic marks related with gene silencing at *CLDN3* promoter region. To address this issue, next we analyzed the enrichment of activating (AcH3 and H3K4me3) and repressive histone marks (H3K9me3 and H3K27me3) at the promoter region of *CLDN3* in LNCaP and abl cells. In abl cells, we observed an increase in the repressive marks and a decrease in the histone active marks compared to LNCaP cells, which correlates with the loss of expression observed in this cell line (Figure 4A). These results could explain, at least in part, the lack of the demethylating treatment effect on CLDN3 expression in abl cells. In fact, the combined treatment with aza and the HDAC inhibitor SAHA increased CLDN3 expression more than the individual treatment (Figure 4B). The tendency of the results showed in Figure 4 strongly indicate that CLDN3 expression is epigenetically regulated in CRPC.

**Figure 5:**
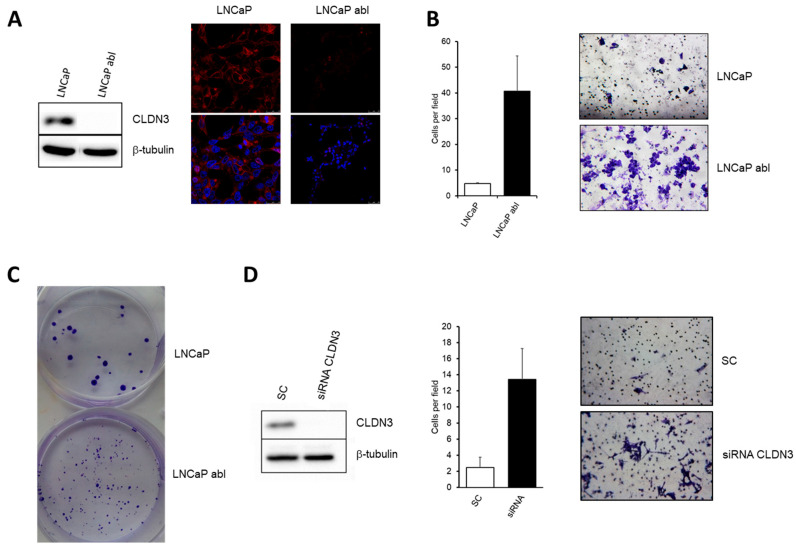
Loss of CLDN3 is associated with increased cellular invasion in prostate cancer cell lines. (**A**) CLDN3 expression in LNCap and LNCaP abl cell lines determined by Western Blot and confocal analysis. Red staining indicates positivity for CLDN3 and nuclei were labelled with DAPI (blue). Micro-photographs were taken at 40X magnification. Scale bar 25 μM (**B**) Comparison of cellular invasion capacity of LNCaP and LNCaP abl cell lines. (**C**) Colony formation assay of LNCaP and LNCaP abl cell lines. (**D**) Effect of CLDN3 siRNA on cellular invasion of LNCaP cells. Loss of CLDN3 is associated with increased invasion capacity of LNCaP cells in a similar way than LNCaP abl compared with LNCaP cells. Micro-photographs were taken at 10X magnification.

To analyze the possible role of CLDN3 in the progression of prostate cancer, we analyzed the effect of CLDN3 loss of expression on the cell phenotype. First, we observed that CLDN3 is expressed by LNCaP cells (Figure 5A), but is not detected in abl cells, which is consistent with the mRNA levels.

The comparison of invasiveness of both cell types showed that invasion capacity of abl was higher than LNCaP cells. (Figure 5B). The number of colonies formed by abl was greater that LNCAP cells, but the cell density of colonies was greater in LNCaP cells (Figure 5C). To confirm if these effects were associated with disturbances in CLDN3 expression, we silenced CLDN3 in LNCaP cells (Figure 5D). CLDN3 knock-down increased cell invasion, which is consistent with the higher invasiveness observed in abl with respect to LNCaP cells.

### 2.3. CLDN3 Expression in Prostate Tumor Samples

**Figure 6:**
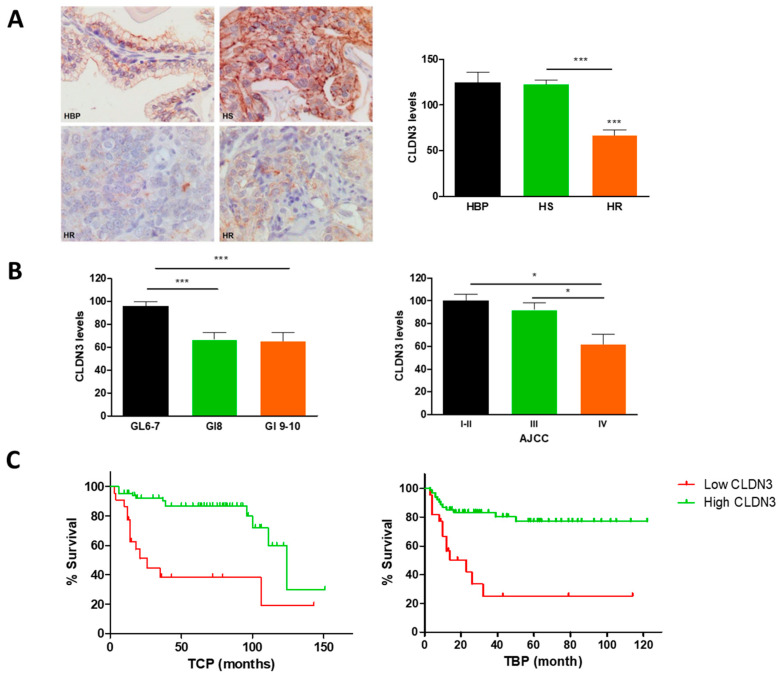
Analysis of CLDN3 expression in normal prostate and prostate cancer samples. (**A**) Micrographs from benign prostatic hyperplasia (BPH), hormone sensitive (HS) and resistant (HR) prostate cancers (PT) (200×). Immunolocalization of CLDN3 (brown staining). The signals for CLDN3 in BPH and HS were intense. In turn, most HR tumors cells showed an evident loss of the signal for CLDN3. The graph shows CLDN3 expression in BPH, HS, and HR prostate tumors measured by immunohistochemistry. (**B**). Analysis of the relationship between CLDN3 levels with Gleason score and AJCC group staging at diagnosis. (**C**) Clinical progression-free survival (TCP) and biochemical progression-free survival (TBP) of patients showing low (red line) or high expression (green line) of CLDN3. * *p* < 0.05; *** *p* < 0.001.

Once the relevance of CLDN3 silencing in a CRPC model was demonstrated, we further investigated CLDN3 expression in samples from patients with benign prostatic hyperplasia (BPH), primary tumors that did not develop resistance to androgen deprivation (androgen-sensitive prostate cancer HS-PC), and tumors that progressed afterwards to CRPC. Prostatic glands from BPH showed high levels of CLDN3 at the cell membranes, and there were no differences with HS-PC, but a significant decrease in CRPC samples was observed (Figure 6A).

Since the samples studied came from a variety of patients with localized, locally advanced and disseminated disease, the relationship between the CLDN3 expression and clinicopathological features of the patients was analyzed. We found a significant decrease in CLDN3 protein levels in samples from patients with high total Gleason score (≥8) and locally advanced tumors (AJCC IV) (Figure 6B). Analyzing clinical follow up yielded CLDN3 loss of expression implied a lower disease-free survival and time to clinical progression (Figure 6C), indicating that CLDN3 expression can predict the prognosis of prostate cancer patients.

## 3. Discussion

Prostate cancer is driven by AR signaling, and several compounds targeting AR pathway and androgen synthesis have been used for advanced prostate cancer treatment. The development of prostate cancer resistance to these treatments remains a universal problem, which ultimately contributes to the deadliness of the disease. Developing treatment resistance can be achieved by AR loss of expression. However, the vast majority of CRPC are characterized by continued AR expression and signaling despite androgen deprivation and AR antagonist treatment. Discrimination between patients either suitable for surveillance strategies or in need of therapy is crucial to prevent over-treatment and to reduce prostate cancer mortality. Uncovering the specific molecular events driven AR-dependent CRPC is crucial for diagnosis and design of specific treatment strategies.

Progression of prostate cancer to castration-resistant and metastatic form of the disease may involve the collaboration of genetic and epigenetic alterations. The most common genetic alteration observed in CRPC is elevated AR copy number and activity [13]. Aberrations in epigenetic modifications, in particular DNA methylation and histone modifications have also been involved in CRPC development. AR regulates gene transcription by its interaction with epigenetic modifiers pointing the focus in a collaborative interaction between genetic and epigenetic alterations in CRPC development.

In this study, we have defined the DNA methylation profile of CRPC cells and identified a panel of epigenetic regulated genes with important roles in CRPC. We found some genes whose methylation changes did not correlate with parallel changes in gene expression. A possible explanation is that those genes might have been transcribed from transcription start sites (TSSs) different from the TSSs that showed the methylation changes. TSSs of a specific gene can vary from tissue to tissue [31], and the TSSs described in the database are not always accurate. Among the selected genes, we demonstrated that reduced expression of CLDN3 was related to promoter hypermethylation in the CRPC cell line abl. Other studies have demonstrated that CLDN3 expression was regulated by promoter methylation in esophageal cancer [32] and ovarian cancer cells [33]. Epigenetic silencing by DNA hypermethylation of other CLDNs have also been described, such as CLDN1 in podocytes [34] and breast cancer [35] and CLDN4 in bladder cancer [36].

CLDN3 belongs to a family of proteins involved in the formation and function of tight junctions. Cell-to-cell adhesion in epithelial cells is maintained through adherent junctions, and changes in the expression of claudins related to these structures have been described in a number of human cancers. However, the expression levels of claudins are heterogeneous and differ even in the same tumor type. In breast cancer, the expression of claudin-1 was found to be downregulated in estrogen receptor (ER)-positive luminal A and luminal B breast cancer, while increased expression was observed in ER-negative basal-like breast cancer subtype [37]. In ovarian cancer, the biological effect of CLDN3 expression appears to be tumor specific. Shang et al., demonstrated that CLDN3 and CLDN4 knock down increase tumor growth and metastatic potential in an ovarian xenograft model [38]. By contrast, other studies have found that CLDN3 silencing reduces cell proliferation and tumor growth [39]. In prostate cancer, CLDN3 expression has been found in normal, PIN, primary and metastatic prostate cancer. Consistent with these studies, we found CLDN3 expression in normal and primary prostate cancer, but it was decreased in patients with advanced prostate cancer that did not respond to hormonal treatments. In addition, CLDN3 loss of expression was correlated with elevated Gleason and shorter disease-free survival and time to clinical progression, strongly suggesting that loss of CLDN3 expression could be an excellent biomarker for the development of the more aggressive forms of the disease.

Despite the number of studies showing the expression of CLDNs in prostate cancer, its biological function is largely unrevealed. It is well accepted that loss of tight junction function is correlated to cancer progression and metastasis [40], since it can cause loss of cell polarity and increased cell invasiveness [41]. Loss of CLDN function in tumors has been suggested to be a mechanism underlying deficient cell adhesion and a crucial event in the evolution of tumor cells toward metastasis [42,43]. In ovarian cancer cells, CLDN3 and CLDN4 loss of expression promotes epithelial-mesenchymal transition (EMT), suggesting a central role in epithelial phenotype [44]. CLDN3 expression inhibits cell motility and invasiveness of hepatocellular carcinoma cells, suggesting a role as metastasis suppressor gene in this tumor type [45]. In this line, our data show that the loss of CLDN3 expression could be preventing cell–cell interactions favoring cell invasion, thus suggesting that the more aggressive phenotype of androgen-independent prostate cancer cells could be explained, at least in part, by CLDN3 epigenetic silencing. Other studies have associated the loss of CLDN3 with decreased cell growth and migration in PC3 cells that represent a model of CRPC lacking AR [46,47]. In the present study, we found CLDN3 downregulated in a model of CRPC expressing AR that represent most of the CRPC patients.

In our study, the loss of CLDN3 expression observed in CRPC cells and tumors could be explained, at least in part, by the loss of CLDN3 control by AR, since it has been demonstrated that AR regulates a different expression profile in androgen-sensitive and independent prostate cancer cells. In Sertoli cells, CLDN3 expression is regulated by AR when stimulated by the canonical pathway (testosterone) through its binding toAREs located at the promoter region of *CLDN3* [48]. Our data show that, in CRPC cells, the loss of CLDN3 expression is associated with an enrichment in epigenetic repressive marks at CLDN3 promoter region, while in the androgen sensitive prostate cancer cells, expressive CLDN3 epigenetic activating marks are present, suggesting that different transcription factors and coregulators are recruited to CLDN3 promoter region in androgen-sensitive and refractory prostate cancer.

We acknowledge certain limitations of our study, such as the lack of animal models to confirm the data from the cell lines, as well as the need to determine the detailed molecular mechanism by which CLDN3 loss of expression is implicated in CRPC progression.

In conclusion, in the present study, we have identified a panel of epigenetic deregulated genes in CRPC to be used as molecular markers for the prediction of CRPC development after androgen deprivation therapy. Of the selected genes, CLDN3 loss of expression is a common event in CRPC that could be used to predict the fall of hormonal response and the development of the more aggressive forms of the disease.

## 4. Materials and Methods

### 4.1. Cell Lines and Tumor Samples

LNCaP, PC3, and DU145 cells were obtained from the DSMZ and maintained in RPMI-1640 with 10% FBS. LNCaP abl cells were provided by Dr. Zoran Culig (Innsbruck Medical University, Austria) [27] and maintained in RPMI-1640 with 10% of charcoal-treated FBS. Cells were maintained at 37 °C in a humidified atmosphere of 95% air and 5% CO_2_. For the treatments, LNCaP and LNCaP-abl cells were treated with 2 μM 5-aza-2-deoxycytidine (Sigma-Aldrich, St. Louis, MO, USA) for 72 h or 5 μM SAHA for 24 h. Treatment was refreshed every 24 h.

CLDN3 expression was evaluated in 89 prostate tumor samples and 10 benign prostatic hyperplasia (BPH) tissues. Disease-free survival analysis was performed with the same cohort from a previous study [24]. Clinical evolution based on PSA and imaging was recorded. The primary endpoint assessed was disease-specific survival. The study was approved by the Ethics Committee of Hospital Universitario de Getafe (A17-11 of 10-27-2011) and was performed in accordance with the ethical standards as laid in the Declaration of Helsinki and its latter amendments.

### 4.2. Methylation450 Beadchip

Genomic DNA isolation was performed according to a standard phenol/chloroform/isoamyl alcohol extraction protocol, after a proteinase K digestion. After bisulfite conversion DNA was used to hybridize on the Infinium HumanMethylation450 Beadchip, following the Illumina Infinium HD Methylation protocol. To calculate the DNA methylation status of the CpG sites, we selected the β-value, which represents the signal ratio from a methylated probe relative to the sum of the methylated and unmethylated probes and takes any value between 0 (unmethylated) and 1 (completely methylated). To avoid possible sources of technical bias that might influence the results, we excluded every β value that had a threshold detection value of *p* > 0.01. GenomeStudio normalizes data using internal controls included in the HumanMethylation 450 BeadChip and also normalizes data with respect to internal back-ground probes.

### 4.3. Bisulfite Genomic Sequencing

For bisulfite genomic sequencing primers were designed using the Methyl Primer Express v1.0 software (Applied Biosystems, Waltham, MA, USA). PCR products were loaded onto 1.5% agarose gels, stained with ethidium bromide and visualized under UV transilluminator. DNA was extracted using QIAquick Gel Extractin Kit and ligated into pGEM-T easy plasmid. The plasmid was transformed in competent bacterial cells and plated onto LB/ampicillin/IPTG/X-Gal plates. A minimum of ten white colonies of each sequence and sample were processed by miniprep and plasmids were sequence automatically to determinate their methylation degree. Primer sequences and annealing temperatures used are available upon request.

### 4.4. Cell Viability, Colony Formation and Invasion Assays

Cell viability was determined by the MTT assay. LNCaP and LNCaP-abl cells were seeded at 1000 cells/well onto 96-well plate, and cell viability was measured at indicated times by staining with MTT and measuring the absorbance at 595 nm. Optical density was directly proportional to cell number up to the maximum density measured.

For colony formation assays, 500 cells/well were seeded into 6-well plates and cultured for 15 days. The colonies were stained with 0.05% crystal violet solution and counted. The experiments were performed at least three times.

Invasion assays were performed in Matrigel chambers (BD). Matrigel invasion chambers were rehydrated with serum-free DMEM or RPMI at 37 °C and 5% CO_2_ for 1 h. Transfected cells were trypsinized, resuspended in serum-free DMEM or RPMI, and 7 × 10^4^ cells were transferred to the upper chamber. 600 µL of growth medium containing 10% FBS was added to the bottom wells. The cells were cultured at 37 °C and 5% CO_2_ for 24 h. Following incubation, the medium was aspired, and the cells remaining on the upper surface of the filter were stained with crystal violet for 1 h. The average number of migrated cells was determined by counting the cells in 3 random high-power field (10×).

### 4.5. mRNA and Protein Analysis

Total RNA was isolated with TRIzol Reagent (Invitrogen, Waltham, MA, USA). An amount of 4 µg of total RNA was reverse transcribed using Super-Script III Reverse Transcriptase (Invitrogen) and using Oligo-dT as primer. PCR amplifications were performed in 96-well optical plates in a volume of 20 μL. We used 0.2 μg of cDNA, 5 pmol of each primer, and 10 μL of 2× SYBRgreen PCR Master Mix (Applied Biosystems). Primers were designed between different exons and encompassing large introns to avoid any amplification of genomic DNA. Primer sequences and annealing temperatures used are available upon request. Expression values were normalized against the expression of glyceraldehyde-3-phosphate-dehydrogenase (GAPDH) and used as an endogenous control to ensure cDNA quality and loading accuracy, following the ΔΔCt method. QRT-PCR was performed on an ABI 7500 Fast (Applied Biosystems, Waltham, MA, USA).

Cell lysates for protein analysis were solubilized in RIPA buffer and resolved through 10% sodium dodecyl sulfate polyacrylamide gels, transferred to a nitrocellulose membrane, and immunoblotted with a polyclonal rabbit anti-CLDN3 (Thermo Fisher, Waltham, MA, USA). Equal loading was tested by reprobing with a polyclonal antibody against human β-tubuline (Sigma). Immunoreactive proteins were visualized by the ECL immunodetection system (Amersham Pharmacia Biotech, Piscataway, NJ, USA) with horseradish peroxidase-conjugated secondary antibodies.

For immunofluorescence assay, cells were fixed with 4% paraformaldehyde, permeabilized in 0.1% TritonX-100 in PBS for 10 min, and stained with antibody against CLDN3 (Thermo Fischer) followed by Alexa Fluor 488-conjugated anti-mouse antibody (Invitrogen). To perform nuclear staining, cells were incubated with DAPI. The fluorescent images were acquired using a confocal microscopy, Leica TCS-SL.

For the analysis of gene expression profile of LNCaP and abl cells GSE11428 dataset was downloaded and differentially expressed genes and volcano map were generated with R Software (v3.2.3).

### 4.6. CLDN3 siRNA

The siRNA targeting CLDN3 was synthesized by Ambion. For this assay, 7 × 10^5^ cells were seeded, and transfection was performed using oligofectamine (Invitrogen, Waltham, MA, USA) according to the manufacturer’s protocol. After 72 h, transfection CLDN3 expression was analyzed by protein blotting. We used Scramble siRNA as a control.

### 4.7. Immunohistochemical Analysis

Paraffin-embedded samples from patients diagnosed of BPH (n = 10) or PC (n = 89) were used. The immunohisto-chemical staining for CLDN3 was performed using a polyclonal rabbit anti-CLDN3 (Thermo Fisher, Waltham, MA, USA), following the method previously described [49]. The immunostaining scores were ranged into four categories based on the staining pattern of the majority of tumor cells in the whole section, which were grouped into two main categories for statistical purposes (0–1: negative/low staining; 2–3: moderate/high staining). Normal human colon samples were used as claudin-immunopositive controls. Negative controls were performed by omitting the first antibody.

Immunostaining scores and clinical data were analysed using one-way ANOVA and or Bonferroni’s multiple comparison tests. Log-rank test and survival curves were used to determine the relationship between claudin-3 staining and time to biochemical and clinical progression. The statistical significance of differences between groups was expressed by asterisks (* 0.01 < *p* < 0.05; ** 0.001 < *p* < 0.01; *** *p* < 0.001).

## Data Availability

The data presented in this study are available upon reasonable request from the corresponding author.

## Supplementary Materials

The following supporting information can be downloaded at: https://www.mdpi.com/article/10.3390/ijms24010803/s1.
