# Supplementary material for: Claudin-3 Loss of Expression Is a Prognostic Marker in Castration-Resistant Prostate Cancer
Source: Int J Mol Sci. 2023 Jan 2;24(1):803. doi: 10.3390/ijms24010803 (PMC9820886; doi:10.3390/ijms24010803)
Supplement: Supplementary file 1 [file ijms-24-00803-s001.zip › Suplementary figure S1.pdf]

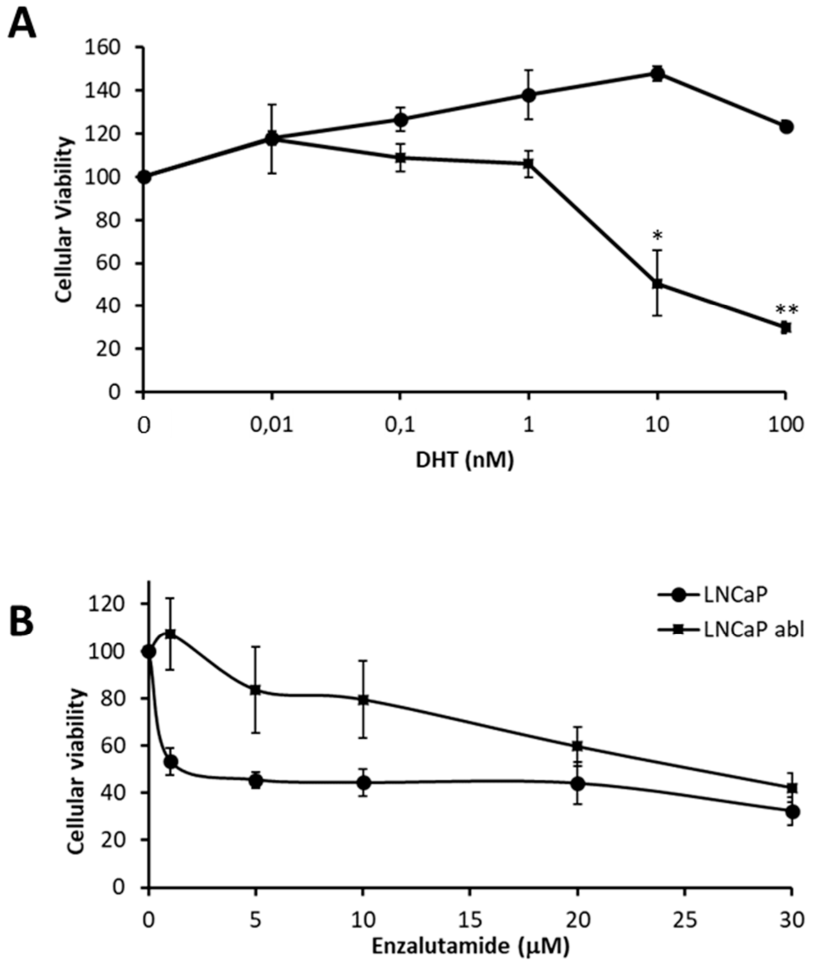

Suplementary figure S1. Effect of Dihydrotestosterone (DHT) and Enzalutamide on cell viability in LNCaP and LNCaP abl cell lines. Cell lines were treated with DHT for 4 days (**A**) or Enzalutamide for 5 days (**B**) at the concentrations indicated and cell viability was monitored by MTT assay. Data shown represent the mean ± s.e.m. of three independent experiments completed in triplicate. * $p < 0.05$; ** $p < 0.01$
