# Supplementary material for: Claudin-3 Loss of Expression Is a Prognostic Marker in Castration-Resistant Prostate Cancer
Source: Int J Mol Sci. 2023 Jan 2;24(1):803. doi: 10.3390/ijms24010803 (PMC9820886; doi:10.3390/ijms24010803)
Supplement: Supplementary file 1 [file ijms-24-00803-s001.zip › Supplementary table S2.pdf]

**Supplementary table S2.** Array DNA methylation values of some of the CpGs analyzed by bisulfite sequencing for CLDN3, EGF and ELF5.

| Gene | CpG position | LNCaP β value | abl β value | Δβ |
|---|---|---|---|---|
| CLDN3 | -70 | 0.02 | 0.49 | 0.47 |
| | -55 | 0 | 0.61 | 0.61 |
| | -41 | 0.05 | 0.69 | 0.64 |
| | -16 | 0.01 | 0.52 | 0.5 |
| EGF | 362 | 0.933 | 0.22 | -0.71 |
| | 551 | 0.92 | 0.18 | -0.74 |
| ELF5 | -266 | 0.926 | 0.337 | -0.549 |
| | -168 | 0.917 | 0.342 | -0.575 |
